# The benefits of cancer screening in kidney transplant recipients: a single‐center experience

**DOI:** 10.1002/cam4.568

**Published:** 2015-12-21

**Authors:** Taigo Kato, Yoichi Kakuta, Toyofumi Abe, Kazuaki Yamanaka, Ryoichi Imamura, Masayoshi Okumi, Naotsugu Ichimaru, Shiro Takahara, Norio Nonomura

**Affiliations:** ^1^ Department of UrologyOsaka University Graduate School of MedicineSuitaJapan; ^2^ Department of UrologyTokyo Women's Medical University Graduate School of MedicineShinjyukuJapan; ^3^ Advanced Technology for TransplantationOsaka University Graduate School of MedicineSuitaJapan

**Keywords:** Cancer screening, immunosuppression, kidney transplantation, posttransplant malignancy

## Abstract

The frequency of malignancy is increasing in kidney transplant recipients. Posttransplant malignancy (PTM) is a major cause of long‐term graft survival inhibition. In this study, we evaluated the frequency and prognosis of PTM at our center and examined the efficacy of cancer screening. Between 1972 and 2013, 750 patients were followed‐up at our center. Annual physical examinations and screenings were performed to detect PTM. We investigated the detail of two distinctive cancer groups: screening‐detected cancers and symptom‐detected cancers. Seventy‐seven PTM were identified during the follow‐up period. The mean age at the initial PTM detection was 43.6 ± 12.8 years. The mean interval from transplantation to cancer diagnosis was 134.5 ± 11.3 months. Among the 77 patients, posttransplant lymphoproliferative disease (PTLD) was the most common cancer (19.5%, 15/77), followed by renal cell carcinoma (15.6%, 12/77). Of the cancer cases, 46.8% (36/77) were detected via screening. The most frequently screening‐detected cancer was renal cell carcinoma of the native kidney and breast cancer (22.2%, 8/36). However, it was difficult to detect PTLD, urothelial carcinoma, and colorectal cancer via screening. Interestingly, Cox proportional regression analyses revealed nonscreened recipients to be a significant prognostic factor for PTM (*P* < 0.001). This study is the first to report that appropriate screening tests play a key role in early PTM diagnosis and lead to reduce the mortality rate in kidney transplant recipients. These findings support the provision of long‐term appropriate screening for kidney transplant recipients.

## Introduction

In the field of kidney transplantation, advances in immunosuppressive agents and transplant surgery have markedly reduced the acute rejection rate and significantly improved graft survival 1. Despite these benefits, the long‐term survival after kidney transplantation has remained unchanged 2, 3. High mortality among kidney transplant patients is attributed mainly to infection as well as malignancy 2, 3. In Asia as well as Europe and North America, chronic immunosuppressant use is associated with an increasing risk of malignancy after kidney transplantation 4, 5, 6. Although the greatest relative increase in this risk has been observed among nonmelanoma skin cancers and oncogenic viral infection‐associated Kaposi sarcoma, the overall trend is directed toward an increased risk of solid organ cancer; the associated relative risk is also significantly increased when compared with the general population 4, 5, 6, 7.

Clinical practice guidelines for the management of posttransplant malignancy (PTM) have been suggested in Europe and North America 2, 8. According to these guidelines, cancer screening should be designed in consideration of the patient and familial history. However, few studies have clearly reported the benefits of screening with regard to reducing the mortality rates associated with gastric and colorectal cancers in kidney transplant recipients 9, 10, 11. In this study, we sought to evaluate the incidence of PTM at our kidney transplantation center and examine the efficacy of screening for monitoring malignant tumors in kidney transplant recipients. Herein, we have demonstrated for the first time that comprehensive cancer screening programs could effectively reduce cancer‐related mortality among kidney transplant recipients.

## Materials and Methods

### Subjects

We followed‐up 750 patients who underwent kidney transplantation at our center between 1972 and 2013. All clinical data for the kidney transplant patients were collected from our departmental database. Among the 750 patients, 77 experienced de novo malignancy (six double cancers). The vast majority of overall patients received a kidney from a living donor (74.3%). The patients' characteristics are shown in Table 1A. Moreover, we divided cancer patients into three groups. Group A patients had screening‐detected cancer. Patients in group B did not undergo screening and patients in group C were not diagnosed with cancer after screening.

**Table 1:** Characteristics of kidney transplant recipients and types of malignancy

(A)		
	Cancer	All transplants
Total number	77	750
Gender		
Male	42	454
Female	35	296
Age of transplantation (years)	43.6 ± 12.8	38.9 ± 10.5
Duration to diagnosis (months)	134.5 ± 11.3	
Duration to dialysis (years)	8.8 ± 2.4	3.7 ± 0.2
Duration of follow‐up (years)	15.1 ± 8.2	14.4 ± 8.6
Number of screening received	56	
Donor source		
Living related	58	557
Cadaveric	14	178
Brain dead	3	15
Immunosuppressant		
Cyclosporine based	33	402
Tacrolimus based	30	314
Conventional	14	34
Pre‐emptive kidney transplantation	6	57
Second transplant	3	8

(B)			
Type of malignancy	Frequency	Gender	
		Male	Female
Lymphomas	15	7	8
Urinary tract			
Renal cell carcinoma of the native kidney	10	9	1
Renal cell carcinoma of the allograft kidney	2	2	0
Urothelial carcinoma	3	1	2
Gastrointestinal tract			
Gastric cancer	8	4	4
Colorectal cancer	5	4	1
Hepatocellular cell carcinoma	3	2	1
Genital tract			
Uterine cancer	3		3
Ovarian cancer	2		2
Breast cancer	9	0	9
Thyroid cancer	6	3	3
Others	11	10	1
Total	77	42	35

In 1993, we introduced routine cancer screening programs for patients. Before 1993, most patients did not undergo screening, although several patients had voluntarily participated in screening tests. The screening test comprised annual abdominal computed tomography (CT) and ultrasonography, chest CT, neck ultrasonography, gastroscopy, and tumor marker tests as well as an annual mammography, breast ultrasonography, and Pap test for female patients. We performed chest and abdominal CT in low dose of radiation without use of contrast media. Skin and lip examinations were performed annually. A fecal occult blood test (FOBT) and urine cytology were performed every 3–6 months. Patients with positive FOBT results were subjected to colonoscopy. If malignancy was suspected during screening, diagnostic studies including tissue biopsy were performed. Once a malignant neoplasm was detected, the patient received appropriate treatment and was followed‐up to assess the tumor response and symptoms. If a patient with malignancy died, cancer was considered the cause of death unless another underlying disease could not be ruled out as a possible cause.

Immunosuppressant therapy largely consisted of a calcineurin inhibitor (CNI), mycophenolate mofetil, and prednisolone. In addition, antilymphocyte globulin (ALG) was used for induction from 1993 to 2003 and was replaced by an anti‐CD25 antibody (basiliximab) beginning in 2004. In patients undergoing ABO‐incompatible renal transplantation, splenectomy or rituximab infusion was performed prior to transplantation. Acute rejection included both biopsy‐proven and clinical rejection cases. Clinical acute rejection was defined as a 20% increase in the serum creatinine level over the baseline. The antirejection therapy protocol included methylprednisolone, ALG, and gusperimus hydrochloride treatment. An anti‐CD3 monoclonal antibody was used for steroid‐resistant rejection. All procedures were performed in accordance with the Helsinki Declaration of 1975. For the purposes of analysis and defining the years of follow‐up, patients were censored at the time of death, PTM diagnosis, the last reported contact, or December 31, 2013.

The study protocol was approved by the Institutional Review Board of Osaka University Hospital (approval number 14150).

### Statistical analysis

The statistical analyses were performed using JMP pro 11.0 software (SAS Institute Inc., Cary, NC). The data on Table 1A are presented as the means ± standard deviations. The level of statistical significance was set at *P* < 0.05. The Kaplan–Meier method was used to calculate the patient survival rates. The primary endpoint was death. Overall survival (OS) was defined as the interval from the time of transplantation to death. Log‐rank tests were used for comparisons between the two groups. Cox regression analyses were used to assess the prognostic factors for PTM.

## Results

### Malignancy

**Figure 1:**
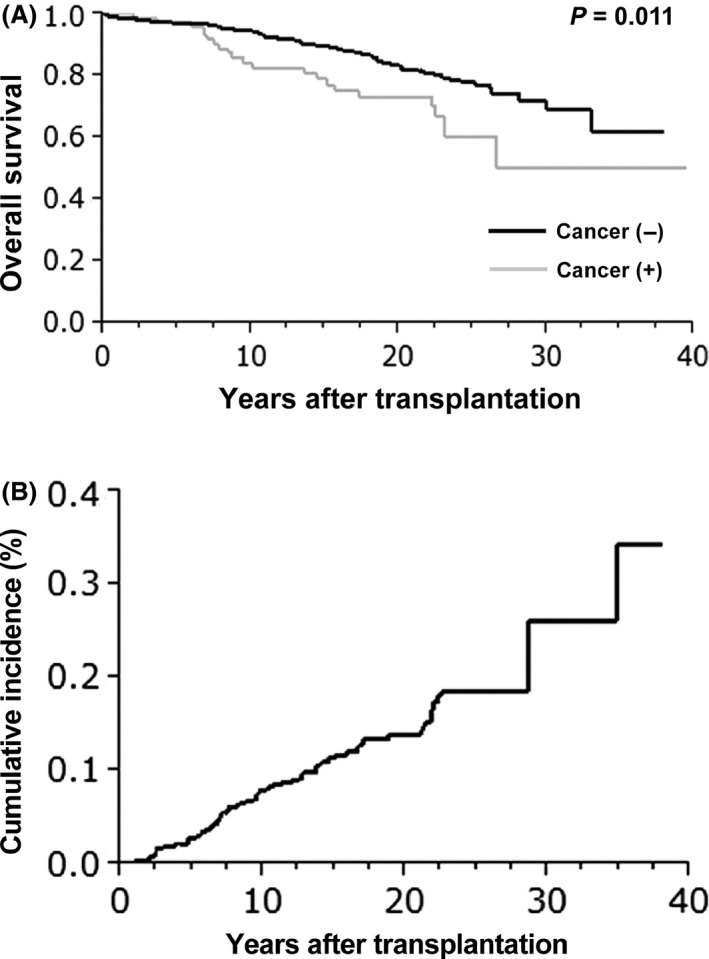
(A) The overall survival of kidney transplant recipients with and without cancer. (B) The cumulative incidence of posttransplant malignancy according to the interval since kidney transplantation.

During the observation period, the overall incidence of malignancy during follow‐up was 10.3% (77/750 patients), with multiple primary malignancies. The mean recipient age was 43.6 ± 12.8 years, and 31.4% of the patients were older than 50 years. The mean interval between transplantation and the time of diagnosis was 134.5 ± 11.3 months. Other patient characteristics are listed in Table 1A. Table 1B lists the observed types of malignancy. Posttransplant lymphoproliferative disorder (PTLD) was the most commonly observed type, representing 19.5% (15/77) of the diagnosed malignancies. Of the 15 cases, six patients were positive for the Epstein–Barr virus (EBV) antibody and one patient was negative before transplantation; the other eight patients were not tested for the EBV antibody. The next most common types were renal cell carcinoma (15.6%, 12/77), breast cancer (11.7%, 9/77), and gastric cancer (10.4%, 8/77). Figure 1A shows a Kaplan–Meier curve of the OS of patients with or without cancer. The respective 5‐ and 10‐year OS rates were 97.2% and 93.9% in the PTM group and 97.3% and 82.6% in the non‐PTM group. Of the 77 patients with PTM, 19 (24.7%) patients died from cancer. The detail in cancer death was three PTLD, three renal cell carcinoma, two uterine cancer, and remaining 11 for various types of cancer. The OS rate in the PTM group was significantly lower than that in the non‐PTM group (Fig. 1A, *P* = 0.011). Figure 1B illustrates the long‐term cumulative PTM incidence rate; this rate was 2.8% at 5 years, 7.8% at 10 years, and 13.8% at 20 years, thereby increasing over time. The 10‐year OS rates of top five PTM were 86.2% in PTLD, 67.5% in renal cell carcinoma, 100% in breast cancer, 80.0% in gastric cancer, and 80.0% in thyroid cancer; besides there is no significant difference between any two groups.

### Cancer detection method and screening

Of the cancer cases, 46.8% (36/77) were detected via screening as shown in Table 2. The most frequently screening‐detected cancer type (group A) was renal cell carcinoma of the native kidney and breast cancer (22.2%, 8/36) and gastric cancer (19.4%, 7/36). Among the patients who did not undergo screening test for various reasons (group B), 9.5% (2/21) developed PTLD and 9.5% (2/21) developed renal cell carcinoma. Cancer patients who were symptomatic at the time of diagnosis but were not detected via screening (group C) largely exhibited PTLD (50.0%, 10/20), urothelial carcinoma (15%, 3/20), or colorectal cancer (15%, 3/20). Patients in groups B and C exhibited cancer‐related symptoms such as central nervous system manifestations with brain PTLD, macrohematuria with urothelial carcinoma, and mucous bloody stools with colorectal cancer. Cox proportional hazard regression analyses were performed to clarify the prognostic factors associated with PTM (Table 3). As shown in Table 3, six prognostic factors were entered into the multivariate analysis, among which nonscreened recipients (*P* < 0.001) and CNI (*P* < 0.001) were found to be significant prognostic predictors of PTM.

**Table 2:** Types of screening‐detected and symptom‐detected cancers after kidney transplantation

	Screening‐detected cancers	Symptom‐detected cancers	
	Group A	Group B	Group C
		Screening (−)	Screening (+)
Lymphomas	3	2	10
Urinary tract			
Renal cell carcinoma of the native kidney	8	1	1
Renal cell carcinoma of the allograft kidney	0	1	1
Urothelial carcinoma	0	0	3
Gastrointestinal tract			
Gastric cancer	7	1	0
Colorectal cancer	1	1	3
Hepatocellular cell carcinoma	1	1	1
Genital tract			
Uterine cancer	2	1	0
Ovarian cancer	1	1	0
Breast cancer	8	1	0
Thyroid cancer	5	0	1
Others	0	11	0
Total	36	21	20

**Table 3:** Cox proportional hazard regression analysis of prognostic factors for post‐transplant malignancy

	Unadjusted hazard ratio	*P* value	Adjusted hazard ratio	*P* value
Male (vs. female)	0.97 (0.56, 1.68)	0.922	1.12 (0.71, 1.77)	0.630
Age ≥ 50 years	1.64 (1.17, 2.50)	0.074	1.18 (0.21, 1.75)	0.056
Nonscreened	2.30 (0.81, 3.75)	<0.001	2.73 (1.06, 3.58)	<0.001
CNI (vs. conventional)	2.45 (1.54, 3.68)	<0.001	1.77 (1.06, 2.84)	<0.001
Cadaveric (vs. living)	1.02 (0.46, 2.03)	0.954	0.99 (0.45, 1.98)	0.993

## Discussion

The incidence of malignancy after kidney transplantation has increased in recent years consequent to increased patient survival 1, 2, 3, 4. Recently, several reports from different countries have compared the incidence of malignancy among kidney transplant recipients with that of the general population 7, 12, 13, 14. In kidney transplantation, the standardized incidence rate (SIR) of all PTM ranged from 3 to 5, although the patterns of malignancy differed among the countries. Many studies conducted in Western countries have reported that skin cancers comprised the most common type of malignancy 7, 12, 14. The Kidney Disease Improving Global Outcomes guidelines recommend daily acitretin administration, especially for patients with a history of skin cancer 8. On the other hand, the incidence of skin cancers was low in Asian countries such as Japan 13, 15, 16. In contrast, gastric cancer, renal cell carcinoma, and PTLD are common in Asian countries.

We analyzed the occurrence and outcomes of posttransplant de novo malignancies in patients who underwent kidney transplantation at our center between 1972 and 2013. The most frequently observed malignancy at our center was PTLD (19.5%), followed by renal cell carcinoma (15.6%), breast cancer (11.7%), gastric cancer (6.5%), and colorectal cancer (6.5%). The incidence of PTLD and breast cancer at our center increased during this 7‐year period 16. The reason for this change was a prolonged follow‐up duration and a committed breast cancer screening test administered to female patients. No skin cancers were observed at our center, in accordance with the low incidence of skin cancers reported in other Asian countries 13, 15, 16.

Several large kidney transplant centers have reported that the cumulative incidence rates of malignancy increase up to 20% after 10 years and 30% after 20 years 17. In contrast, at our center the cumulative incidence rates of malignancy were 7.8% after 10 years and 13.8% after 20 years. These low cumulative incidence rates might have resulted from the intense medical screening that comprised part of the pretransplantation evaluation process. Moreover, we attempted to sufficiently moderate the administered CNI concentration and thus minimize the PTM incidence.

Clinical physicians face the difficulty of balancing PTM progression and reducing the immunosuppressant dose, the latter of which may increase the risk of allograft impairment. Given this difficulty, physicians should attempt to avoid the unnecessary incidence of PTM through malignancy prevention and early detection. Nearly half of all PTM cases at our center were detected via appropriate screening. The most frequently screening‐detected cancers were breast cancer (8/9 patients) and gastric cancer (7/9 patients). The 5‐year survival rates of these cancers were 100% and 88.9%, respectively. Transplant recipients who develop breast cancer are often younger at diagnosis and have poorer outcomes relative to the general population 2, 18. Considering these facts, intense screening for both cancer types is profoundly significant in terms of early‐stage malignancy detection and appropriate treatment.

On the other hand, the number of PTLD cases has increased during this 7‐year period, particularly in group C. Among PTLD cases, brain PTLD (4/10) is especially difficult to detect because the screening process does not cover regular brain CT. Moreover, chest and abdominal CT are poorly able to detect gastrointestinal PTLD 19. Urinary cytology also has a low specificity for urothelial carcinoma detection. At our center, no cases of urothelial carcinoma were detected via urinary cytology. Colorectal cancer is also difficult to detect because of the low FOBT false‐negative rate 8. Currently, we are faced with the necessity of developing new strategies for identifying these difficult‐to‐detect cancers. For example, positron emission tomography (PET)‐CT might be an option for detecting PTLD and colorectal cancer because of its high detection rate 20. Future trials should be extensively evaluated to clarify the benefits of PET‐CT for PTM.

Cancer screening has been proven effective for reducing the cancer‐related mortality and morbidity in the general population, but unfortunately randomized controlled trials of screening tests have not been conducted in the context of transplantation 8, 21. Although some cancers were difficult to detect, in our study noncancer screening had a significantly high hazard ratio, which confirmed the benefits of screening. To the best of our knowledge, this study demonstrated for the first time that in kidney transplant recipients, comprehensive cancer screenings could reduce the mortality rate and improve the recipients' prognoses. These findings support the provision of long‐term appropriate screening for kidney transplant recipients.

From the perspective of PTM prevention, a recent concept is that mammalian target of rapamycin inhibitors (mTORis) could reduce the frequency of PTM 7, 22. Recent studies suggest that the use of the mTORis sirolimus and everolimus might reduce the overall risk of de novo solid cancer in patients who have undergone kidney transplantation 7, 23. Beginning in 2010, our center replaced CNI with everolimus in patients with stable renal function. We await the outcome of this change and the possible protective effect of everolimus, specifically a reduced frequency of malignancy among kidney transplant recipients.

Some limitations should be considered when interpreting these results. First, the longer follow‐up period might have allowed us to detect more cancer cases, especially late‐onset cases. Second, some cancer cases in the early study period might have been underreported because of deficient clinical data. Finally, this was a retrospective study and some relevant clinical information might have been limited.

In conclusion, given an appropriate screening program kidney transplant recipients with PTM might be detected during an early disease stage, receive treatment using minimally invasive modalities, and have an excellent evolution. Although the sample size was small, this study has important implications, particularly that cancer screening programs are needed to improve the outcomes of kidney transplant recipients. On the basis of our findings, we propose careful cancer screening in kidney transplant recipients.

## Conflict of Interest

None declared.
